# Risk of Job Loss During the COVID-19 Pandemic Predicts Anxiety in Women

**DOI:** 10.3390/medicina61020178

**Published:** 2025-01-21

**Authors:** Nina Krohne, Tina Podlogar, Vanja Gomboc, Meta Lavrič, Nuša Zadravec Šedivy, Diego De Leo, Vita Poštuvan

**Affiliations:** 1 Slovene Centre for Suicide Research, Andrej Marušič Institute, University of Primorska, 6000 Koper, Slovenia; tina.podlogar@upr.si (T.P.); vanja.gomboc@iam.upr.si (V.G.); meta.lavric@iam.upr.si (M.L.); nusa.sedivy@iam.upr.si (N.Z.Š.); diego.deleo@upr.si (D.D.L.); 2 Department of Psychology, Faculty of Mathematics, Natural Sciences and Information Technologies, University of Primorska, 6000 Koper, Slovenia

**Keywords:** COVID-19, women, employment insecurity, anxiety, mental health

## Abstract

*Background and Objective*: During the COVID-19 pandemic, women faced unique employment-related stressors, including higher exposure to unstable working conditions, increased workload changes due to motherhood, and greater risk of infection in certain jobs. This study explores how these factors influence women’s anxiety and subjective well-being, aiming to identify vulnerable groups. *Materials and Methods*: 230 employed Slovene women, aged from 19 to 64 years (M = 32.60, SD = 10.41), participated in an online survey containing a State-Trait Anxiety Inventory (STAI-6), WHO-5 Well-being Index, and a set of questions regarding their occupation and demographic profile. Hierarchical linear regressions and chi-squared tests were performed. *Results*: The risk of job or income loss significantly predicted an increase in anxiety levels. However, despite fear of infection, none of the work-related variables predicted a significant decrease in subjective well-being. Women reporting risk of job or income loss are predominantly those with lower education and income, working students, self-employed, or working in the private sector. *Conclusions*: Employment insecurity is an important contributor to anxiety in women. The findings highlight the need to ensure job security, particularly for women working in precariat working conditions, as their work and economic stability prove to be vulnerable to external economic disturbances.

## 1. Introduction

The global pandemic of COVID-19 led affected countries, including Slovenia, to adopt protection measures and safety precautions to contain the spread of the infection [1]. Slovenia recorded its first case of infection with the SARS-Cov-2 virus on 4 March 2020 [2]. Following the first case, the number of positive cases increased exponentially [2], resulting in the proclamation of an epidemic on 12 March 2020 [2]. The proclamation was followed by a nationwide lockdown, instructing the public to strictly follow social distancing, self-isolate, and remain within their households [2]. Schools closed, and many public and private organizations had to discontinue or adapt their industrial and service activities. By the end of March 2020, a fifth of employed Slovenes were furloughed, approximately 10% were on leave, and around 4% reported having already lost their employment. Of those remaining at work, 9% reported working less, and 20% reported increased workload [3].

The inevitable socio-economic implications of protective measures caused concerns about a consequent economic crisis [4]. Slovene legislation mandates employers to provide partial financial support to employees who are unable to work or are furloughed [5]. Due to mandatory business closure, many companies and organizations could not maintain the liquidity required to financially support their employees, so the Slovene government introduced aid measures aimed at mitigating the economic impact of the crisis [6].

Certain groups, including women, might be more susceptible to the negative consequences of the pandemic, including employment security [7]. Studies consistently display that during the pandemic, women have a higher risk of unemployment than men [8,9,10], which is particularly salient in young women [11]. Nevertheless, young age and female gender seem to be important risk factors for precariousness [12]. Researchers [12,13] recognize that this is not due to individual preferences but rather due to rising economic disparities and a shift toward labor market flexibility, resulting in a growing number of low-skill jobs and part-time employment, which often leave young people in temporary or unstable jobs without security or benefits. Additionally, the disparity between the skills acquired through education and those demanded by the labor market results in an educational-employment mismatch [14], forcing young people to occupy positions for which they are overqualified. For women, these challenges are further compounded by gender inequalities, including discrimination and unequal access to stable employment [15,16]. These combined factors make young women particularly vulnerable in today’s labor markets, especially in times of crisis.

Dang and Nguyen [9] showed that women across six countries in Asia, Europe, and America, were 24% more likely than men to permanently lose their jobs during the COVID-19 outbreak. In Slovenia, at the beginning of 2020, before the lockdown, the number of registered unemployed people was greater in men. In the first months of lockdown, the number of unemployed people increased for both genders; however, the increase was larger in women [17]. The vast majority of unemployed persons were waiters [18], a profession dominated by women [19].

The experience of losing a job is a particularly stressful event associated with numerous mental health issues, including an increase in depression and anxiety [20]. Watson and Osberg [21] suggested that, due to uncertainty, the risk of job loss might potentially cause greater psychological distress than actual job loss. They further emphasized that job insecurity affects mental health through subjective factors, such as an individual’s perception of their job stability, and objective factors, including measurable risks of joblessness determined by individual attributes and macroeconomic conditions. Vulnerable populations, including working students, are often faced with a conjoint effect of personal insecurities stemming from experiencing economic hardship and job insecurities associated with flexible labor markets in the neoliberal era [13]. Pandemic-related work or income instability, thus, only adds to the pre-existing struggle.

When assessing the role of employment in the mental status of female employees, losing a job might not be the only factor contributing to a decline in women’s mental health during the pandemic. In the UK, women are disproportionately represented in occupations associated with a high risk of infection, such as frontline roles in healthcare and caregiving [22]. Similarly, in Slovenia, women frequently serve as frontline workers in medical settings and retail environments, both of which carry an elevated risk of infection [19].

During the lockdown, many employees experienced changes in their working hours, either working fewer hours due to temporary job discontinuation or caregiving responsibilities, or working more due to increased demands and work overload. Kleppa et al. [23] indicate that increased workloads are associated with heightened anxiety and depression, as working overtime introduces additional stressors [24] while reducing time for leisure and family interactions [23]. Conversely, working fewer hours may improve well-being due to increased leisure time. However, it may also signal employment instability, causing concern, and disrupting working routines, potentially impairing one’s sense of coherence and increasing anxiety [25]. Nevertheless, with school closures, many mothers did not benefit from increased leisure time. Instead, they replaced paid work hours with unpaid domestic labor, including childcare, eldercare, and household responsibilities [26].

Generally, women display a higher tendency to develop anxiety-related disorders compared to men [27]. This was reflected in the psychological assessments during the first wave of the pandemic; women, in contrast to men, reported increased levels of stress, anxiety, depression, and trauma [28,29], causing a substantial concern for their mental health status [30]. A large study [31] investigating the global prevalence and burden of COVID-19-related depressive and anxiety disorders, which included 204 countries and territories, further highlighted this disparity, showing that women experienced a disproportionately higher burden of depressive and anxiety disorders during the pandemic. This heightened vulnerability was attributed to several factors, including increased caregiving responsibilities and greater economic insecurity, reflected in job insecurity, lower wages, and reduced savings. These findings underscore the need to address the gender-specific impacts of mental health during a pandemic.

Our study aims to explore how various work-related factors, such as fear of losing employment, changes in workload, and risk of infection, impact anxiety and subjective well-being among women. Women are more likely than men to experience anxiety and are disproportionately affected by unstable working conditions, many of which have been exacerbated by the COVID-19 pandemic. Additionally, women often face unique challenges related to changes in workload due to motherhood and are overrepresented in positions with higher risks of infection. This study seeks to address this issue by examining the intersection of gender, employment, and mental health.

## 2. Materials and Methods

This study was part of a larger research project aimed at assessing the mental health status during the first COVID-19 lockdown. In this section, we describe the methodological information concerning the research objective of the present study—an association between employment-related conditions during the first COVID-19 lockdown and women’s mental health. Additional aspects of the research project are described in other publications [32].

### 2.1. Procedure

Data were collected through an online survey at the time of the very first lockdown in Slovenia, between 26 March and 7 April 2020. Employing a convenience sampling technique, we shared the invitation containing a link to the survey through various websites, emails, and social media channels. The only inclusion criterion was being 18 years old or older.

The participants responded to the invitation by clicking the link to the survey. The survey was administered through a Slovenian survey platform 1ka.si (University of Ljubljana, Ljubljana, Slovenia). When visiting the survey, participants were informed about the study’s aims and procedure, potential risks, and sources of support in case of distress. They were presented with informed consent, highlighting that their participation is voluntary and anonymous, allowing them to discontinue it at any time. They were asked to consent to their data being analyzed on a group level and disseminated to wider audiences. The consent was given by clicking a button, allowing them to access the survey (see Section 2.3). The survey took approximately 20 min to complete.

### 2.2. Participants

Altogether, 259 women completed the survey; however, the unemployed and the retired were excluded from the analysis. This resulted in a final sample of 230 women aged between 19 and 64 years (M = 32.60, SD = 10.41). This indicates that the sample is rather young. None of the participants reported having COVID-19. Additional demographic characteristics of the sample are presented in Table 1.

The demographic profile of the sample, illustrated in Table 1, indicates that compared to the general population of women in Slovenia [19], more women who participated in this study come from western Slovenia regions and urban areas. Additionally, more of them completed some tertiary education and are employed in the public sector or working students. For most of them, their income does not reach the average net monthly rate, which was 1.146, 11 EUR [19] at the time of conducting the study. Most women are childless, which might be due to the generally young age of the sample.

### 2.3. Measures

A larger battery of self-administered questionnaires and questions was used for the study. The battery aimed to assess various aspects of mental health and well-being. Below, we describe the selected instruments used in the present study. All instruments were administered in the Slovene language.

State-Trait Anxiety Inventory (STAI-6) [33] for State Anxiety was used to measure feelings of anxiety from the time of the official declaration of the COVID-19 pandemic in Slovenia (12 March 2020) to the day of completing the survey. Participants rate their sensations on a four-point Likert-type scale from 1 (not at all) to 4 (very much so). The final score ranges between 6 and 24; high scores indicate experiencing high levels of anxiety. A six-item measure is a short form [33] of the original 20-item State-Trait Anxiety Inventory, Form X [34]. The short form demonstrated acceptable reliability and validity, yielding results comparable to the full form [33]. The latter underwent a reliability generalization study [35], including 816 research articles using STAI between 1990 and 2000, indicating acceptable reliability coefficients for internal consistency and test-retest reliability. In the present study, the value of Cronbach’s alpha demonstrated excellent internal consistency (see Table 2).

WHO-5 Well-Being Index [36] was used to measure subjective well-being from the time of the official declaration of the COVID-19 pandemic and the first lockdown in Slovenia (12 March 2020) to the day of completing the survey. The measure consists of five items, employing a six-point Likert-type scale from 0 (at no time) to 5 (all of the time). The final score ranges between 0 and 25; high scores indicate a high level of subjective well-being. A validation study based on item response theory and measurement invariance involving 35 countries concluded that WHO-5 is a psychometrically sound measure of subjective well-being, demonstrating satisfactory reliability [37]. The latter was confirmed by a high value of Cronbach’s alpha in the present study (see Table 2).

The participants were presented with the following work-related questions, employing dichotomous (yes or no) answers: “Do you work (or study) in an environment with a high risk of getting infected with the coronavirus SARS-CoV-2 (e.g., usually implying the presence of other people)?”, “Do you risk losing your job (or income) due to the COVID-19 epidemic?” and “Did you lose your job (or income) due to the COVID-19 epidemic?”. Furthermore, participants were presented with the question, “What changes, related to the quantity of your work, did you experience due to the COVID-19 epidemic?”, with three possible answers, namely (1) working less, (2) working more, or (3) working the same amount as before. Lastly, a question concerned changes in working environment or conditions (e.g., working from home or inability to perform their work).

Participants were asked to rate the level of fear they felt regarding becoming infected with the SARS-CoV-2 virus on a 10-point Likert-type scale from 0 (not afraid at all) to 10 (very afraid).

Lastly, participants were asked to provide information regarding their gender, age, employment status, education, income, region, type of residence (urban vs. rural), and number of people in their household.

### 2.4. Data Analysis

We used IBM SPSS Statistics (version 25) software (IBM, SPSS Inc., Chicago, IL, USA) to perform the analyses. Firstly, we inspected the descriptive statistics and explored the reliability of the measures by calculating Cronbach’s alpha. Subsequently, we designed the regression models, utilizing the STAI-6 and WHO-5 scores as outcome variables and the employment-related variables as predictive variables. In parallel, we examined the compliance of our data with the linear regression’s assumptions. Lastly, we observed the frequencies regarding certain socio-occupational characteristics of the group facing a risk of job or income loss and calculated the chi-square values with post hoc tests to further specify the risk groups. Education and income variables were dichotomized.

## 3. Results

Circa one-third of the participants felt the risk of losing their employment or income due to the pandemic (*n* = 71, 31%). A total of 15 participants (7%) reported having already lost their job. Slightly more than one-third of participants reported a risk of contracting the SARS-CoV-2 virus at their workplace (*n* = 87, 38%). Considering possible changes in the quantity of work during the pandemic, the majority of participants reported working less (*n* = 91, 40%), whilst about one-third of them reported working more (*n* = 69, 30%) or not experiencing any changes in the quantity of work (*n* = 67, 29%). The descriptive statistics for the numerical variables are presented in Table 2.

Data in Table 2 reveal that the participants’ scores covered the entire spectrum of final scores for the STAI-6 measure and Fear of Infection; however, none of the participants reached the highest score (25 points) on the WHO-5 measure. Considering the standard deviations, we can observe a non-zero variance for all the measures, indicating the ability to perform regression analysis [38]. Skewness and kurtosis values suggest minimal deviation from normality, ensuring the validity of linear regression assumptions [38]. Both STAI-6 and WHO-5 display excellent internal consistency, as evidenced by high Cronbach’s alpha values.

The data complied with all the assumptions for linear regression [38], allowing for its computation. We present the results of our regression models in Table 3. We constructed the models to assess whether job loss or a risk of job (or income) loss, risk of infection at work, and the changed quantity of working time (working less or more) predict feelings of anxiety (STAI-6) and subjective well-being (WHO-5). Additionally, we controlled for fear of infection and monthly income, which may affect the outcomes.

In Table 3 we see that the controlled variables—general fear of infection with SARS-CoV-2 and low income—explain 20% of the variance for state anxiety and 13% for subjective well-being. The variance is predominantly explained by fear of infection, which significantly increases feelings of anxiety and decreases subjective well-being.

Adding the employment-related variables to the model did not result in a significant increase in the share of explained variance, indicating a rather small role of employment-related factors in explaining anxiety and subjective well-being. However, employment-related variables in both models make better predictors of anxiety and subjective well-being as opposed to using the mean value (F(7, 193) = 7.78, *p* < 0.001 for STAI-6 and F(6, 225) = 4.48, *p* < 0.001 for WHO-5).

The risk of job or income loss has a significant role in explaining anxiety, regardless of employment-related factors. As shown in Table 3, women who report feeling this risk, presented a score 1.56 points higher on the STAI-6 on average, compared to women who do not perceive this risk.

Considering the important role of pandemic-related risk of job or income loss in predicting higher levels of anxiety, we inspected the occupational profile of women, who are particularly exposed to this risk. The results revealed the risk is reported by 70% of self-employed women (*n* = 12), 50% of women who work in the private sector (*n* = 15), and 47% of students (*n* = 35). On the contrary, this risk was reported only by 8% of women who work in the public sector (*n* = 9). A chi-square value of 51.65 and post hoc tests suggest that these differences are significant (*p* < 0.01 for all pairings).

The results furthermore revealed that women who perceive the risk of income loss are mostly those whose workload decreased due to the pandemic (45% reported the risk, *n* = 41). Those whose amount of work did not change appear to feel the lowest risk of job or income loss, with only 13% (*n* = 9) reporting the risk. A chi-square value of 18.50 and post hoc tests suggest these differences are significant (*p* < 0.001 for both pairings). Moreover, we observed that most women who declared that they could not work during the pandemic and did not receive monetary supplements (*n* = 27, 84%) feel the risk of job or income loss (χ^2^ = 57.05, *p* < 0.001).

With regard to socio-economic status, the risk is expressed by 43% of women (*n* = 56) whose monthly income is below the average (χ^2^ = 19.71, *p* < 0.001). Additionally, women who report the risk are predominantly women with secondary education or less (54%, *n* = 29, χ^2^ = 16.80, *p* < 0.001). The results suggest that, during the pandemic, higher social status—reflected in higher monthly income and higher educational attainment—serves as a protective factor against perceiving the risk of job or income loss.

## 4. Discussion

The present study aimed to investigate the role of employment-related factors, including job/income loss, risk of job/income loss, risk of infection at the workplace, and altered working time, in predicting women’s anxiety and subjective well-being during the first lockdown in Slovenia (spring 2020). The results revealed that employment or income uncertainty is a notable stressor contributing to anxiety levels, independent of other factors such as income level or fearing infection. The negative impact of job or income insecurity on mental health has been frequently reported, as well as an increase in anxiety-related symptoms when facing the situation of employment insecurity [20]. None of the factors significantly predicted a decrease in subjective well-being. Thus, the results suggest that during the first wave of the COVID-19 pandemic, women who perceived the risk of job or income loss felt significantly more worried and fearful, while their feelings of happiness, liveliness, and energy levels were not significantly affected by the risk.

Most women facing the risk of job or income loss worked under unstable employment conditions, including student work, self-employment, or private-sector employment. A temporary state of heavily disrupted or completely shut down business may cause worries about the long-term stability of these workplaces, while public sector institutions may be less vulnerable to the influence of an economic crisis, particularly in times of recession [39]. Moreover, the participants facing losing their employment or income are women with lower education and lower monthly income, who were not able to perform their jobs during the pandemic, nor did they receive any monetary supplement for their working inability. This profile of women facing employment instability is consistent with findings from other studies [11] and they might be at higher risk of experiencing feelings of employment- or income-related anxiety during the pandemic.

The findings contribute to the body of research on the adverse effects of precarity and gender-specific challenges, emphasizing the importance of ensuring job stability for women during crises. In Slovenia, the government addressed the need to support unstable employment by providing financial aid to certain groups, including those in the private sector, the self-employed, working parents, and students [6,40]. However, the aid was tied to specific parameters (e.g., the share of lost income), which excluded some individuals from eligibility. Moreover, the monetary compensation for certain groups, including students, was minimal, potentially forcing those reliant on student jobs to return to their hometowns, thereby impacting their academic progress and personal development. In Slovenia, student work differs from regular employment and is governed by distinct organizations and legal frameworks. As a result, working students are often ineligible for standard work-related legal protections, leaving this group particularly vulnerable to economic instability.

April 2020 saw a significant rise in unemployment rates for both genders, followed by fluctuations in response to lockdown measures [17]. Interestingly, in 2020, the gender pay gap in Slovenia dropped substantially [41]. Unemployment began to decrease in spring 2021, with this trend continuing throughout 2022, 2023, and 2024 [17]. By the end of 2024, Slovenia’s unemployment rate had reached historic lows [17], surpassing pre-pandemic levels and signaling improved job stability across the population. Despite this recovery, certain groups likely faced substantial challenges during the initial stages of the pandemic. These findings underscore the critical importance of timely and targeted measures to mitigate hardship among vulnerable populations and protect their mental health.

The crisis measures alone may not be sufficient to enhance (young) women’s ability to withstand economic disturbances. It is essential to implement policies that systematically address the structural barriers women face in the labor market. In Slovenia, the years following 2020 saw a gradual increase in the gender pay gap [41], reflecting heightened wealth inequality between genders. Women might be at greater risk of occupying lower-paid positions despite pursuing higher education, as reflected in the demographic characteristics of the sample in this study. These circumstances place women in a particularly vulnerable position, especially in the context of potential future pandemic-related or other economic challenges. As highlighted by OECD [42], the recommended policies include promoting paternity leave, pay transparency for equal pay, flexible but safe work opportunities, and representation of women in leadership roles. Additionally, gender equality considerations should be integrated into broader policy areas, such as trade, energy, and transport, while addressing systemic barriers like gender stereotypes, discrimination, and underrepresentation in policymaking.

The high predictive value of the fear of infection, predicting increased anxiety and decreased subjective well-being, pinpoints the importance participants ascribe to their health and the level of danger they attribute to the virus. Nevertheless, in a public survey performed in Slovenia, 44% of Slovenes expressed concerns regarding their health in the first week following the announcement of the pandemic and lockdown [3]. Oreffice and Quintana-Domeque [22] found that compared to men, women report higher concerns for infection.

The amount of variance explained by employment-related predictors included in the regression models is relatively low. Thus, there might be other factors influencing the psychological functioning of women during the COVID-19 pandemic. Nevertheless, a decrease in mental health amidst pandemic-related circumstances has been frequently reported by mental health professionals [43,44]. Various reasons have been identified, including social isolation, social distancing, quarantine, caregiver stress, death/illness, and unemployment [45]. It is also possible that employment-related factors and employment instability are not of high significance for women. A study from Germany revealed that during the pandemic, men expressed a higher concern for paid work and the economy, whereas women worried more about childcare [45].

This study is not without limitations. Firstly, a convenience sampling technique was adopted, which decreases the representativeness of the sample. It includes an overrepresentation of younger women, women from western Slovenia and urban areas, and women with higher education. The data concerning specific demographic variables, including ethnic background, mental health or disability status, being a single parent, being a career, etc., are missing. Consequently, we are refraining from generalizing the results to the general population of women in Slovenia and suggesting a closer examination of the mental status of the specific vulnerable groups of women. Secondly, the present study is cross-sectional, allowing for bidirectional connections between the variables. This is particularly relevant to the fear of infection; it is possible that people who feel more anxious also experience more fear of infection as a result of their anxiety. Thirdly, the employment-related variables were assessed through one item only, which can reduce the reliability of the findings. Lastly, the STAI-6 measure used in the present study has been validated in English; only the original 20-item STAI (Form X) measure has been validated in the Slovene language.

## 5. Conclusions

This study revealed that a risk of job or income loss due to the COVID-19 pandemic is an important employment-related factor, significantly impairing women’s psychological functioning by increasing their feelings of anxiety. Considering the sample included in the study, including a high proportion of young, highly educated women from urban areas in western Slovenia with below-average personal income, the effect cannot be generalized to the general population of women in Slovenia. The risk of job or income loss predominantly affected women of lower educational and economic status who are either working students, self-employed, or working in the private sector. Combined with additional stressors, including health concerns, increased burden of domestic tasks [26], and potential exposure to domestic violence [30], employment insecurity, embedded in the organizational structures of the patriarchal and neoliberal labor architectures, might be an additional factor impacting women’s mental health during the first lockdown of the COVID-19 pandemic.

The mental health ramifications of employment instability might extend beyond the COVID-19 pandemic. Despite declining unemployment rates, the post-pandemic era has introduced new economic stressors, including inflation [46], which has diminished individuals’ purchasing power and reduced their ability to save. Additionally, recent years have seen an increase in the gender pay gap [41], further limiting women’s capacity to mitigate the effects of economic crises and potentially exacerbating pandemic-related mental health challenges. Thus, the results of the present study highlight the need for greater economic security for women working in precariat employment conditions, as they seem to be most vulnerable to external economic disturbances.

## Figures and Tables

**Table 1:** Demographic characteristics of the sample.

		f	%	Missing (f)
Region				1
	Eastern Slovenia	84	36.52	
	Western Slovenia	144	62.61	
Type of Residence				1
	Urban	143	62.17	
	Rural	86	37.39	
Education				1
	Basic education	2	0.87	
	Secondary education	53	23.04	
	Tertiary education	174	75.65	
Employment				0
	Employed in public sector	107	46.52	
	Employed in private sector	30	13.04	
	Self-employed	17	7.39	
	Working student ^a^	76	33.04	
Personal monthly net income ^b^				2
	Below 1280 EUR	168	69.57	
	Above 1280 EUR	68	29.57	
Children				4
	No	144	62.61	
	Yes	82	35.65	
Change in work quantity due to COVID-19				3
	No changes	67	29.13	
	Working less than before COVID-19	91	39.57	
	Working more than after COVID-19	69	30.00	
Monetary compensation for not being able to work due to COVID-19 ^c^				178
	No	32	13.91	
	Yes	20	8.70	

Note. The total sample size (N) consists of 230 participants, and the percentages (%) are calculated based on this sample size. f = frequency. ^a^ Students who perform part-time student jobs managed by specific agencies and legal frameworks. ^b^ The average net monthly wage in Slovenia when collecting the data (April 2020) was 1.146,11 EUR [19]. ^c^ Only the participants who reported being unable to work due to COVID-19 are included.

**Table 2:** Descriptive statistics for numerical variables.

	Min–Max	M (SD)	Skewness (SE)	Kurtosis (SE)	Cronbach’s α
STAI-6	6–24	14.41 (4.35)	0.39 (0.16)	−0.67 (0.32)	0.91
WHO-5	0–24	12.70 (5.17)	−0.32 (0.16)	−0.42 (0.32)	0.89
Fear of infection	0–10	5.01 (2.26)	−0.02 (0.17)	−0.39 (0.34)	

Note. n_STAI-6_ = 230, n_WHO-5_ = 230, n_Fear_ = 201. STAI-6 = State Trait Anxiety Inventory for state anxiety. WHO-5 = Well-being Index.

**Table 3:** Results of hierarchical regression analyses.

		STAI-6			WHO-5		
Variable		B	SE (B)	β	B	SE (B)	β
Block 1							
	Fear of infection	0.86	0.13	0.43 ***	−0.84	0.16	−0.36 ***
	Low income	0.83	0.59	0.09	−0.33	0.73	−0.03
R^2^			0.20			0.13	
F for change in R^2^ (2, 191)			23.11 ***			14.45 ***	
Block 2							
	Fear of infection	0.85	0.13	0.43 ***	−0.84	0.16	−0.36 ***
	Low income	0.50	0.63	0.05	−0.27	0.77	−0.03
	Risk of job (or income) loss	1.56	0.73	0.16 *	−0.70	0.90	−0.06
	Job loss	0.38	1.37	−0.02	−0.07	1.70	−0.01
	Risk of infection at work	0.06	0.63	0.01	−0.04	0.78	−0.04
	Working less	−0.57	0.75	−0.06	0.71	0.93	0.07
	Working more	0.53	0.75	0.05	−0.65	0.93	−0.06
R^2^			0.23			0.14	
F for change in R^2^ (5, 186)			1.75			0.56	

Note. *n* = 194. STAI-6 = State-Trait Anxiety Inventory for state anxiety. WHO-5 = Well-being Index. B = unstandardized beta. SE (B) = standardized error for unstandardized beta. β = standardized beta. * *p* < 0.05, *** *p* < 0.001.

## Data Availability

The data used in the study are available at University of Primorska, Andrej Marusic Insitute, Slovene Center for Suicide Research. COVID-19 and Mental Health; 26 March to 7 April 2020, Slovenia. Ann Arbor, MI: Inter-university Consortium for Political and Social Research [distributor], 26 February 2021. https://doi.org/10.3886/E132981V1.

## References

[1] Legal Information System of the Republic of Slovenia Display of Applicable Regulations Adopted to Prevent the Spread of COVID-19 Disease. http://www.pisrs.si/Pis.web/aktualno#.

[2] COVID-19 Pandemic in Slovenia. https://en.wikipedia.org/wiki/COVID-19_pandemic_in_Slovenia.

[3] Zadnji Ukrepi Vlade za Zajezitev Širjenja Virusa Sprejeti Precej Negativno, Dobra Tretjina Zaposlenih Trenutno Delovno Neaktivnih [The Latest Governmental Measures to Contain the Spread of the Virus Received Negatively, One Third of the Employees Inactive]. https://www.valicon.net/sl/2020/04/zadnji-ukrepi-vlade-za-zajezitev-sirjenja-virusa-sprejeti-precej-negativno-dobra-tretjina-zaposlenih-trenutno-delovno-neaktivnih/.

[4] Nicola M., Alsafi Z., Sohrabi C., Kerwan A., Al-jabir A. (2020). The socio-economic implications of the coronavirus pandemic (COVID-19): A review. Int. J. Surg..

[5] (2013). Zakon o Delovnih Razmerjih, ZDR-1 [Employment Act, ZDR-1]. Official Gazette of the Republic of Slovenia, no. 21/13, 78/13—Popr., 47/15—ZZSDT, 33/16—PZ-F, 52/16, 15/17—Odl. US, 22/19—ZPosS, 81/19, 203/20—ZIUPOPDVE, 119/21—ZČmIS-A, 202/21—Odl. US, 15/22, 54/22—ZUPŠ-1, 114/23 in 136/23—ZIUZDS. https://pisrs.si/pregledPredpisa?id=ZAKO5944.

[6] (2020). Zakon o Interventnih Ukrepih za Zajezitev Epidemije COVID-19 in Omilitev Njenih Posledic za Državljane in Gospodarstvo, ZIUZEOP [Act on Intervention Measures to Contain the COVID-19 Epidemic and Mitigate Its Consequences for Citizens and the Economy, ZIUZEOP. Official Gazette of the Republic of Slovenia, No. 003-02-03/2020-2. https://www.uradni-list.si/glasilo-uradni-list-rs/vsebina/2020-01-0766/zakon-o-interventnih-ukrepih-za-zajezitev-epidemije-covid-19-in-omilitev-njenih-posledic-za-drzavljane-in-gospodarstvo-ziuzeop.

[7] Burki T. (2020). The indirect impact of COVID-19 on women. Lancet Infect. Dis..

[8] Alon T., Doepke M., Olmstead-Rumsey J., Tertilt M. (2020). This Time It’s Different: The Role of Women’s Employment in a Pandemic Recession. Natl. Bur. Econ. Res..

[9] Dang H.A.H., Nguyen C.V. (2021). Gender inequality during the COVID-19 pandemic: Income, expenditure, savings, and job loss. World Dev..

[10] Gezici A., Ozay O. (2020). An Intersectional Analysis of COVID-19 Unemployment. J. Econ. Race Policy.

[11] Moen P., Pedtke J.H., Flood S. (2020). Disparate disruptions: Intersectional COVID-19 employment effects by age, gender, education, and race/ethnicity. Work Aging Retire.

[12] Kretsos L., Tremmer J. (2010). The persistent pandemic of precariousness: Young people at work. A Young Generation Under Pressure? The Financial Situation and the “Rush Hour” of the Cohorts 1970–1985 in a Generational Comparison.

[13] Rydzik A., Bal P.M. (2024). The age of insecuritisation: Insecure young workers in insecure jobs facing an insecure future. Hum. Resour. Manag. J..

[14] Büchel F. (2002). Successful apprenticeship-to-work transitions: On the long-term change in significance of the German school-leaving certificate. Int. J. Manpow..

[15] OECD (2019). Part-Time and Partly Equal: Gender and Work in The Netherlands, Gender Equality at Work.

[16] Stamarski C.S., Son Hing L.S. (2015). Gender inequalities in the workplace: The effects of organizational structures, processes, practices, and decision makers’ sexism. Front. Psychol..

[17] Employment Service of Slovenia Registrirana Brezposelnost [Registered Unemployment]. https://www.ess.gov.si/partnerji/trg-dela/trg-dela-v-stevilkah/registrirana-brezposelnost/.

[18] Employment Service of Slovenia Vpliv Epidemije SARS-CoV-2 na trg dela v Sloveniji [The Influence of SARS-CoV-2 Epidemic on the Labour Market in Slovenia]. https://www.ess.gov.si/fileadmin/user_upload/Trg_dela/Dokumenti_TD/Analize/Analiza_vpliva_epidemije_SARS-CoV-2_na_trg_dela.pdf.

[19] Statistical Office of the Republic of Slovenia SiStat Database [Data Set]. https://pxweb.stat.si/SiStat/en.

[20] Goldman-Mellor S.J., Saxton K.B., Catalano R.C. (2010). Economic contraction and mental health: A review of the evidence, 1990–2009. Int. J. Ment. Health.

[21] Watson B., Osberg L. (2018). Job insecurity and mental health in Canada. Appl. Econ..

[22] Oreffice S., Quintana-Domeque C. (2020). Gender Inequality in COVID-19 Times: Evidence from UK Prolific Participants.

[23] Kleppa E., Sanne B., Tell G.S. (2008). Working overtime is associated with anxiety and depression: The Hordaland Health Study. J. Occup. Environ. Med..

[24] Spurgeon A., Harrington J.M., Cooper C.L. (1997). Health and safety problems associated with long working hours: A review of the current position. Occup. Environ. Med..

[25] Uchida H., Tsujino D., Muguruma T., Hino N., Sasaki K., Miyoshi M., Koyama Y., Hirao K. (2018). Low sense of coherence is associated with anxiety among adults: Results based on data from all 47 prefectures of Japan. Compr. Psychiatry.

[26] Almeida M., Shrestha A.D., Stojanac D., Miller L.J. (2020). The impact of the COVID-19 pandemic on women’s mental health. Arch. Womens Ment. Health.

[27] McLean C.P., Anderson E.R. (2009). Brave men and timid women? A review of the gender differences in fear and anxiety. Clin. Psychol. Rev..

[28] Wang C., Pan R., Wan X., Tan Y., Xu L., Ho C.S., Ho R.C. (2020). Immediate Psychological Responses and Associated Factors during the Initial Stage of the **2019** Coronavirus Disease (COVID-19) Epidemic among the General Population in China. Int. J. Environ. Res. Public Health Artic..

[29] Guadagni V., Umilta’ A., Iaria G. (2020). Sleep Quality, Empathy, and Mood During the Isolation Period of the COVID-19 Pandemic in the Canadian Population: Females and Women Suffered the Most. Front. Glob. Women’s Health.

[30] Thibaut F., van Wijngaarden-Cremers P.J.M. (2020). Women’s Mental Health in the Time of COVID-19 Pandemic. Front. Glob. Women’s Health.

[31] COVID-19 Mental Disorders Collaborators (2021). Global prevalence and burden of depressive and anxiety disorders in 204 countries and territories in 2020 due to the COVID-19 pandemic. Lancet.

[32] Lavrič M., Gomboc V., Krohne N., Podlogar T., Poštuvan V., Zadravec Šedivy N., De Leo D. (2020). Concerns, Positive Changes, and Suggestions for Psychological Support during COVID-19: A Thematic Analysis. Sociol. Mind..

[33] Marteau T.M., Bekker H. (1992). The development of a six-item short-form of the state scale of the Spielberger State—Trait Anxiety Inventory (STAI). Br. J. Clin. Psychol..

[34] Spielberger C.D., Gorsuch R.L., Lushene R.E. (1970). Manual for the State-Trait Anxiety Inventory.

[35] Barnes L.L., Harp D., Jung W.S. (2002). Reliability generalization of scores on the Spielberger state-trait anxiety inventory. Educ. Psychol. Meas..

[36] World Health Organisation (WHO) (1998). Wellbeing Measures in Primary Health Care/The Depcare Project.

[37] Sischka P.E., Costa A.P., Steffgen G., Schmidt A.F. (2020). The WHO-5 well-being index–validation based on item response theory and the analysis of measurement invariance across 35 countries. JAD Rep..

[38] Field A. (2017). Discovering Statistics Using IBM SPSS Statistics.

[39] Kopelman J.L., Rosen H.S. (2015). Are Public Sector Jobs Recession-proof? Were They Ever?. Public Financ. Rev..

[40] Vlada Sprejela #Proti-Koronapaket [Government Adopts #Anti-Coronapackage]. https://www.gov.si/novice/2020-03-29-vlada-sprejela-protikoronapaket/.

[41] SDG Indicators, Goal 5 Achieve Gender Equality and Empower All Women and Girls. https://www.stat.si/Pages/en/goals/goal-5.-achieve-gender-equality-and-empower-all-women-and-girls/5.1-gender-pay-gap.

[42] OECD (2023). Joining Forces for Gender Equality: What Is Holding Us Back?.

[43] Lima C.K.T., de Medeiros Carvalho P.M., Lima I.D.A.A.S., de Oliveira Nunes J.V.A., Saraiva J.S., de Souza R.I., da Silva C.G.L., Neto M.L.R. (2020). The emotional impact of Coronavirus 2019-nCoV (new Coronavirus disease). Psychiatry Res..

[44] Sritharan J., Sritharan A. (2020). Emerging Mental Health Issues from the Novel Coronavirus (COVID-19) Pandemic. J. Health Med. Sci..

[45] Czymara C.S., Langenkamp A., Cano T. (2020). Cause for concerns: Gender inequality in experiencing the COVID-19 lockdown in Germany. Eur. Soc..

[46] Hafner M. (2024). Kratke Analize: Vpliv Rasti cen Hrane na Inflacijo [Brief Analyses: The Impact of Rising Food Prices on Inflation].

